# Knowledge, Attitude, Practice, and Status of Infection Control among Iranian Dentists and Dental Students: A Systematic Review

**DOI:** 10.5681/joddd.2013.010

**Published:** 2013-05-30

**Authors:** Behnam Moradi Khanghahi, Zahra Jamali, Fatemeh Pournaghi Azar, Mohammad Naghavi Behzad, Saber Azami-Aghdash

**Affiliations:** ^1^Medical Philosophy and History Research Center, Tabriz University of Medical Sciences, Tabriz, Iran; ^2^Dental and Periodontal Research Center, Tabriz University of Medical Sciences, Tabriz, Iran; ^3^Assistant Professor, Department of Oral Medicine, Faculty of Dentistry, Tabriz University of Medical Sciences, Tabriz, Iran; ^4^Assistant Professor, Department of Operative Dentistry, Faculty of Dentistry, Tabriz University of Medical Sciences, Tabriz, Iran; ^5^Tabriz Health Services Management Research Center, Faculty of Management and Medical Informatics, Tabriz University of Medical Sciences, Tabriz, Iran

**Keywords:** Attitude, infection control, knowledge, practice, systematic review

## Abstract

**Background and aims:**

Infection control is an important issue in dentistry, and the dentists are primarily responsible for observing the relevant procedures. Therefore, the present study evaluated knowledge, attitude, practice, and status of infection control among Iranian dentists through systematic review of published results.

**Materials and methods:**

In this systematic review, the required data was collected searching for keywords including infection, infection control, behavior, performance, practice, attitude, knowledge, dent*, prevention, Iran* and their Persian equivalents in PubMed, Science Direct, Iranmedex, SID, Medlib, and Magiran databases with a time limit of 1985 to 2012. Out of 698 articles, 15 completely related articles were finally considered and the rest were excluded due to lake of relev-ance to the study goals. The required data were extracted and summarized in an Extraction Table and were analyzed ma-nually.

**Results:**

Evaluating the results of studies indicated inappropriate knowledge, attitude, and practice regarding infection control among Iranian dentists and dental students. Using personal protection devices and observing measures required for infection control were not in accordance with global standards.

**Conclusion:**

The knowledge, attitudes, and practice of infection control in Iranian dental settings were found to be inadequate. Therefore, dentists should be educated more on the subject and special programs should be in place to monitor the dental settings for observing infection control standards.

## Introduction

Infection is a major problem for health care systems in many countries.^1^ Infection control has gained much attention especially in dentistry after introduction of HIV infection in 1980s, and reports of contamination of six patients by a dentist.^2^ Among health care professionals, dentists are more prone to infection due to their direct contact with blood and saliva on a daily basis in their offices.^3^ In spite of advances in infection control in recent years, there is still infection control problem in health care centers including dentistry clinics and hospitals.^4,5^

Although several recommendations and guidelines are issued by medical and dental societies as well as governmental organizations, studies demonstrate that infection is not well-controlled in the dental settings and hospitals.^6^ The results of previous studies indicate inappropriate knowledge, attitude, and practice regarding proper measures of infection control among dentists.^7-9^ Studies conducted in Iran also demonstrate a lower-than-standard knowledge, attitude, and practice among dentists regarding the issue.^10-12^ There has been, however, no systematic approach to summarize the results of such studies to enable offering a comprehensive, yet clear viewpoint of the current status of infection control in the dental profession in Iran.

Therefore, the present study aimed to systematically summarize and report the results of studies evaluating infection control measures in the dental settings as well as knowledge, attitude, and practice of dental professionals regarding infection control.

## Materials and Methods

**Figure 1:**
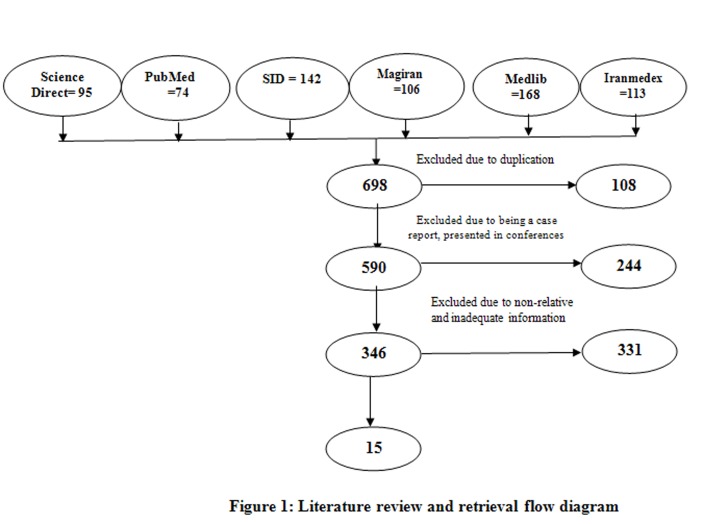
Literature review and retrieval flow diagram.

In this systematic review study, the required data was collected searching for keywords including infection control, Iran, behavior, attitude, knowledge, dent*, infection prevention and their Persian equivalents through PubMed, Science Direct, SID, Medlib, Magiran, and Iranmedex databases. Articles published between 1985 to 2012, articles which dealt with infection control or dentists’ knowledge, attitude and practice, studies that collected the data using questionnaires and observation, and articles published in Persian and English were regarded as inclusion criteria of the study. Letters to editor, case reports, and articles presented at conferences were excluded from the study. Following database searching, authentic journals in Persian and English were hand searched in order to identify and cover more published articles. After excluding those articles with weak relationship to the study objectives, references of the selected articles were re-searched to ascertain identification and evaluation of all available literature and find more related articles. Out of 698 articles, 15 completely-related articles were finally selected and the rest with weak relationship to the study objectives were excluded (Figure 1). The selected articles were evaluated meticulously. Three articles were in English. Nine articles dealt with dentists’ knowledge, attitude, and practice on infection control. The required data were summarized in an extraction table and analyzed manually.

## Results

### Dentists’ Knowledge, Attitude, and Practice on Infection Control

Self-reported scores of dentists’ knowledge, attitude, and practice on infection control are summarized in Table 1. Where other indices were used in a study, they were converted to percentage in order to facilitate comparisons.

Approximately 60% of dentists demonstrated appropriate practice regarding infection control (moderate interval regarding classifications of studies about practice on infection control). The results showed dispersion and deviation regarding dentists’ knowledge about required measures of infection control, as the rate varied from 35% to 75%. The knowledge of dentists on infection control was overall about 50%. A positive attitude toward infection control was seen among dentists (>70%).

### Applying Staff Protection Devices among Dentists

This study reported four most important and necessary devices used in infection control. The rate of staff protection devices was included in ten articles (Table 2). The rate varied significantly among dentists from 7% in using glasses to 98% in using a cover.

### Necessary Actions in Infection Control among Dentists

Nine articles had studied the rate of observing principles and measures required for infection control. Table 3 summarizes the results. Several studies reported measures such as washing hands and applying disinfectants. Similar to the rate of using staff protection devices, there were differences between the rate of observing necessary actions towards infection control (33% for washing hands to 98% for applying disinfectants).

**Table 1:** Table 1. Dentists’ and dental students’ knowledge, attitude, and practice regarding infection control

Author	Year	City	Practice score	Knowledge score	Attitude score
Askarian & Assadian^11^	2003	Shiraz	55.22 ± 24.11	74.55 ± 11	77.75 ± 9.93
Ebrahimi et al^13^	2009	Mashhad	68 ± 8	34.9 ± 13	—
Zakerijafari & Mohammadisalimi		Rasht	62.2(weak), 37.8(moderate), 0 (good)	1.1 (weak), 24.4 (moderate), 74.5 (good)	1.1 (weak), 54.4 (moderate), 44.4 (good)
Eghbal et al^14^	2002	Tehran	58.37	52.85	—
Simari et al^15^	2001- 2002	Tehran	—	1.8 (weak), 25.3 (moderate), 68.8 (good), 4.1(excellent)	15.5 (weak), 73.2 (moderate), 11.3 (good)
Ajami et al^10^	2008	Mashhad	27.5(weak), 60.4(moderate), 12.1 (good)	35 ± 13.3	—
Mohammadrazavi et al	2003	Isfahan	68.25 ± 14.7	—	—
Hekmatian & Khalfi, ^16^	2012	Boushehr	—	45	—
Alipour et al^17^	2005	Bandarabbas	69 ± 6.55	54 ± 8.8	—

**Table 2:** Application rate of staff protection devices among dentists and dental students

Study/ personal protection devices	City	Year	Gloves (%)	Glasses (%)	Mask (%)	Cover (%)
Askarian & Assadian	Shiraz	2003	82.9	51.3	76.3	30.3
Ebrahimi et al	Mashhad	2009	97.95	65.30	97.95	—
Eghbal et al	2002	37.3	27.3	—	—
Geramipanah & Monzavi	Varamin		76.9	64	—	—
Najafi Dolatabadi et al^18^	Yasouj	2005-2006	93	60	90	90
Mohammadrazavi et al	Isfahan	2003	69.4	44.9	82.1	92.5
Hashemipour et al^19^	Kerman	2007	23.3	7.7	15.5	36.9
Alavian et al^20^		2004	93.1	—	—
Najafi Dolatabadi et al ^2^	Yasouj	2005-2006	97	78	92	98
Zakerijafari & Mohammadisalimi^21^	Rasht	-	82.2	28.9	82.2	95.6

**Table 3:** Measures reported for infection control in the reviewed studies

Study/infection control measures	Washing hands	Disinfection of equipment	Separation of infectious waste material	Environment health	Disinfectants & sterilizers	Disposable devices	Hepatitis vaccination
Askarian & Assadian / 2003 - Shiraz	45.4	—	—	—	—	—	—
Geramipanah & Monzavi / Varamin	96.2	91	—	—	97.9	95.7	—
Najafi Dolatabadi et al / 2005- 2006 - Yasouj	43	73	—	—	93	68	—
Ajami et al / 2008- Mashhad	33.1	—	—	—	—	89.9
Mohammadrazavi et al / 2003 - Isfahan	>70	>70	—	>50 70>	50>	52	93.4
Hashemipour et al / 2007 - Kerman	—	—	—	—	42.7	—	52.4
Alavian et al / 2004	—	—	—	—	—	—	74.8
Najafi Dolatabadi et al ^2^/ 2005- 2006 - Yasouj	82	95	—	—	92	97	—
Valiollahi et al^22^/ 2004 - Tehran	—	—	53.47	56.55	97.29	—	—
Alipour et al/ 2005 - Bandarabbas	56.7	74	61	81	67	—	24.7

## Discussion

Taking measures required for infection control is an important part of daily dental practice from both patients’ and practitioners’ point of view. In this regard, it is necessary to have a good knowledge of the required measures and a positive attitude, as well as the appropriate practices in place. Evaluating the results of those studies conducted in Iran indicate a relatively low level of knowledge, attitude, and practice regarding infection control among dentists and dental students. Also, the results suggest that using personal protection devices and taking necessary measures required for infection control are not in accordance with global standards.

The results of the studies evaluated in this review demonstrate that Iranian dentists’ and dental students’ knowledge, attitude, and practice are on an overall poor level. Accordingly, outcomes of other studies conducted in countries such as United States, Italy, Nigeria, and England refer to low level of dentists’ knowledge, attitude, and practice.^7,8,23,24^ Therefore, there seems to be a need for more education and emphasis regarding infection control for dental students and dentists. Additionally, providers of dental services and supervising organizations should focus more on the subject.

The obtained results suggest that rate of using personal protection devices among Iranian dentists for infection control is not at a desirable level. For example, although it is recommended to use gloves during all procedures involving contact with the patient, this is not always observed by about 25% of the dentists. The rate of using gloves and cover is higher than that of glasses and mask. Given the above-mentioned cases, glasses are used less than other three devices, and a significant gap between the current status and the ideal condition. In comparison with other countries, it can be noted that gloves are used more often in other countries than in Iran. One study conducted in Jordan, for instance, demonstrated that about 81% of the dentists wear gloves while performing dental procedures.^1^ Another study among orthodontists in Illinois, US showed gloves are used in 97% of the cases. The figure is about 94% in a Canadian study.^25^ In comparison with high income countries, the use of other personal protection aids such as masks, covers, and glasses is at a lower level among the Iranian professionals. Montagna et al^26^ have reported 96%, 91%, and 92% as application rate of gloves, mask, and eye-protection glasses. The statistics demonstrate a high application rate of personal protection devices in comparison with Iranian dentists. However, some of previous studies show varying rates of using personal protection devices by dentists^27-30^ This indicates the need for further continuing education with emphasis on using personal protection devices.

Findings of the present study indicate an inappropriate level of observing necessary actions for infection control such as washing hands before and after contact with the patients, using disposable items, applying disinfectants, and hepatitis B vaccination among Iranian dentists. It can be inferred while a relatively acceptable status exists for measures like using disposable items and disinfectants, other measures like separation of infectious wastes from non-infectious waste, environment health, and washing hands are in a relatively undesirable status. A national study conducted in Canada demonstrated that 94-100% of dentists used disposable devices and 60-96% of them disinfected handpieces after every application.^25^ The results indicate that 25-94% of Iranian dentists are vaccinated against hepatitis B. This rate varies between 68%-98.9% in different countries,^31-34^ which indicates a better condition in comparison with Iran.

The findings of the presents study demonstrated a relatively desirable condition for disinfection of dental equipments (exceeding 70%). Another study has shown while about 89% of dentists have the necessary equipments to disinfect their devices, only 45% of them use them.^6^ The rate of observing infection control measures were shown to be at a low level among Iranian dentists. The issue has been seen in several other studies too.^1,35,36^

The findings of the studies evaluated have been reported in diverse forms and it can be regarded as a weak point for the present study, not allowing for quantitative analyses and calculating of results mean. However, this study attempted to offer a comprehensive viewpoint towards infection control among Iranian dentists and dental students through summarizing and reporting different aspects of infection control. In order to evaluate the conducted studies and to improve the condition of infection control, further intervention studies are recommended.

## Conclusion

Evaluating the results of previously conducted studies indicated an inappropriate knowledge, attitude, and practice among Iranian dentists and dental students regarding infection control. The rate of using personal protection devices and observing necessary procedures for infection control was not consistent with the accepted standards. Therefore, education should be provided and infection control subjects should be emphasized as a priority of academic curricula. Additionally, special programs should be in place to monitor the dental settings for observing infection control standards.
